# Impact of central nervous system (CNS) prophylaxis on the incidence of CNS relapse in patients with high-risk diffuse large B cell/follicular grade 3B lymphoma

**DOI:** 10.1007/s00277-020-04140-0

**Published:** 2020-06-23

**Authors:** Hanne Kuitunen, Elina Kaprio, Peeter Karihtala, Ville Makkonen, Saila Kauppila, Kirsi-Maria Haapasaari, Milla Kuusisto, Esa Jantunen, Taina Turpeenniemi-Hujanen, Outi Kuittinen

**Affiliations:** 1 Medical Research Center, Department of Oncology and Radiotherapy, Oulu University Hospital, University of Oulu, Kajaanintie 50, P.O. Box 5000, 900l4 Oulu, Finland; 2 Cancer and Translational Medicine Research Unit, University of Oulu, Kajaanintie 50, P.O. Box 5000, 900l4 Oulu, Finland; 3 Cancer Center, Kuopio University Hospital, Puijonlaaksontie 2, 70210 Kuopio, Finland; 4 Comprehensive Cancer Center, University of Helsinki and Helsinki University Hospital, P.O. Box 100, FI-00029 Helsinki, Finland; 5 The North Karelia Central Hospital, Tikkamäentie 16, 80210 Joensuu, Finland; 6 Medical Research Center, Department of Pathology, Oulu University Hospital, University of Oulu, Kajaanintie 50, P.O. Box 5000, 900l4 Oulu, Finland; 7 Department of Medicine, Kuopio University Hospital, Puijonlaaksontie 2, 70210 Kuopio, Finland; 8 Faculty of Health Medicine, Institute of Clinical Medicine, University of Eastern Finland, Kuopio, Finland

**Keywords:** Diffuse large B cell lymphoma, Central nervous system prophylaxis, High-dose methotrexate, Central nervous system recurrence

## Abstract

Although overall survival in diffuse large B cell lymphomas (DLBCL) has improved, central nervous system (CNS) relapse is still a fatal complication of DLBCL. For this reason, CNS prophylaxis is recommended for patients at high risk of CNS disease. However, no consensus exists on definition of high-risk patient and optimal CNS prophylaxis. Systemic high-dose methotrexate in combination with R-CHOP has been suggested as a potential prophylactic method, since methotrexate penetrates the blood-brain barrier and achieves high concentration in the CNS. In this retrospective analysis, we report treatment outcome of 95 high-risk DLBCL/FL grade 3B patients treated with R-CHOP or its derivatives with (*N* = 57) or without (*N* = 38) CNS prophylaxis. At a median follow-up time (51 months), CNS relapses were detected in twelve patients (12.6%). Ten out of twelve (83%) of CNS events were confined to CNS system only. Median overall survival after CNS relapse was 9 months. Five-year isolated CNS relapse rates were 5% in the prophylaxis group and 26% in the group without prophylaxis. These findings suggest that high-dose methotrexate-containing prophylaxis decreases the risk of CNS failure.

## Introduction

Diffuse large B cell lymphoma (DLBCL) is the most frequent form of non-Hodgkin lymphoma, accounting for 25–58% of all NHL cases [1, 2]. DLBCL is responsive to chemotherapy and with current immunochemotherapy regimen, R-CHOP or its derivatives, about 60% of patients achieve long-term disease-free survival [3–5]. The addition of rituximab (R) to cyclophosphamide, doxorubicin, vincristine and prednisolone (CHOP) chemotherapy has improved the response rate and the overall survival in DLBCL patients. However, simultaneously with improving systemic disease control, solitary central nervous system relapses have evolved as an increased problem. Patients with disease progression into the CNS have poor survival, despite aggressive interventions, with median survival of about 2–5 months [6–8]. The incidence of CNS recurrence varies from 5 to 25% in DLBCL [8–14]. After facing this problem in Oulu University Hospital, we incorporated into our treatment algorithm an intravenous high-dose methotrexate (MTX)-based CNS prophylaxis to patients at high risk of CNS relapse according to evaluation by clinical risk factors.

With this background, we report on a retrospective analysis of 95 high-risk DLBCL patients treated with R-CHOP, R-CEOP or R-CHOEP immunochemotherapy with or without CNS prophylaxis during 2006–2012, mainly in Oulu University Hospital. We compared these results with patients with similar risk profile treated without MTX-containing prophylaxis and who were either treated in other hospitals, with different local treatment algorithms, or had contraindications to systemic high-dose methotrexate. We report the incidence and risk factors for CNS relapse and impact of CNS prophylaxis on CNS relapse rates.

## Material and methods

### Patients, staging and treatment

Patients had a high-risk DLBCL or follicular lymphoma grade 3B, were diagnosed between January 2006 and December 2012 and treated at our hospital, except for seven patients treated in Kuopio University Hospital where the patients were treated without CNS-targeted therapy in line with the local protocol. Patients with human immunodeficiency virus (HIV) or CNS involvement at diagnosis were excluded. Two patients were excluded from the analyses due to decreased general condition and death soon after the prephase therapy.

Baseline clinical characteristics including WHO performance status (PS) between grades 0 and 4, routine chemistry profiles, clinical stage with a whole-body computer tomography (CT) and bone marrow aspiration and biopsy, number and type of extranodal site and International Prognostic Index (IPI) and CNS-IPI scores were recorded [15, 16]. Stage was defined in accordance with the Ann Arbor system [17]. The histopathological samples were reviewed and if possible, cases were subclassified as germinal centre B cell-like (GCB) and non-GCB in accordance with the Hans algorithm [18]. The retrospective study was performed according to the Declaration of Helsinki and approved by the ethics committee of Oulu University Hospital, Finland.

Of the 104 high-risk DLBCL patients and one FL grade 3B patient diagnosed, 103 patients received at least one cycle of R-CHOP or its derivatives with curative intent. Eight patients (of 103) received only intrathecal methotrexate as CNS prophylaxis and were excluded from the analysis. *N* = 27 (28%) patients were treated with R-CHOP, *n* = 43 (45%) patients received R-CEOP and 25 (26%) patients were treated with R-CHOEP. Median number of cycles were correspondingly R-CHOP, 7 (range 6–8); R-CEOP, 6 (range 1–8); and R-CHOEP, 7 (range 5–9) cycles. The median follow-up time was 49 (range 0–110) months.

### CNS prophylaxis

The screening for CNS involvement at diagnosis was at the discretion of the treating physician and was performed if the patient had any neurological signs or symptoms. However, for 55% of the patients who received CNS prophylaxis, the lumbar puncture (LP) had been performed before the initiation of systemic therapy. In this situation, CNS involvement was excluded by neuroimaging with magnetic resonance imaging (MRI) of the brain or cerebrospinal fluid (CSF) examination with cytology and/or flow cytometry. The high risk of CNS relapse was defined by any of the following: high-risk International Prognostic Index score (IPI) ≥ 3, elevated LDH and more than one extranodal site or specific extranodal sites being sinus, epidural, testicular and breast. We also calculated CNS-IPI, consisting of the individual IPI factors and involvement of renal and/or adrenal glands for a total of six factors.

In line with the treatment algorithm at Oulu University Hospital, all high-risk patients were treated with CNS-targeted therapy with high-dose methotrexate from 3 to 5 g/m^2^ simultaneously with MTX IT therapy on day 1 after R-CHOP or its derivatives infusion. Correspondingly, patients in Kuopio University Hospital were treated with R-CHOP. In elderly patients, the dose of i.v. methotrexate was according to the discretion of the treating physician. The number of MTX cycles varied in accordance with tolerability from 1 to 3 cycles, and were typically administered with systemic immunochemotherapy cycles 1–3 or 2–4. The main reason to combine CNS prophylaxis with cycle 2–4 was pre-treatment thirdspace fluid as ascites. The HD-MTX dose and sequence and the number of cycles were at the treating physicians’ discretion.

Based on actualized or unfulfilled CNS prophylaxis, the patients were separated into three risk groups corresponding to the different strategies of CNS-targeted therapy (Table 1):

**Table 1:** Three risk groups corresponding to the different strategies of CNS-targeted therapy, based on actualized or unfulfilled CNS prophylaxis

Prophylaxis group	CNS prophylaxis regimen		R-CHOP	R-CEOP	R-CHOEP
	Intravenous HD-MTX	Intrathecal MTX			
1 (*n* = 43)	3–5 g/m^2^ × 2–3	12.5 mg × 2–3	10	13	20
2 (*n* = 14)	1–3 g/m^2^ × 1–3	12.5 mg × 1–3	1	11	2
3 (*n* = 38)	None	None	16	19	3
Median number of cycles (and range)			7 (6–8)	6 (1–8)	7 (5–9)

*R-CHOP*, rituximab-cyclophosamide-doxorubicin-vincristine-prednisone; *R-CEOP*, rituximab-cyclophosamide-epirubicin-prednisone; *R-CHOEP*, rituximab-cyclophosamide-doxorubicin-vincristine-etoposide-prednisone

Group 1: Intravenous HD-MTX 3–5 g/m^2^ × 2–3 + IT MTX 12.5 mg × 2–3 and the minimum cumulative dose of MTX ≥ 9 g/m^2^,

Group 2: Intravenous HD-MTX 1 – 3 g/m^2^ × 1–3 + MTX 12.5 mg IT × 1–3.

Group 3: Patients did not receive CNS-targeted therapy.

### Response evaluation and follow-up

The response was evaluated in accordance with the International Working Group response criteria [19] and, after 2007, following the revised International Working Group response criteria [20]. Response to therapy was evaluated after four, six and eight courses and thereafter every three months for two years (whole-body imaging twice a year) and then every six months until five years from the treatment (whole-body imaging once a year). Neuroimaging was only performed for patients with neurological symptoms or deficits. The diagnosis of CNS recurrence was confirmed by MRI and by CSF cytology or flow cytometry. Simultaneous system relapse was excluded by whole-body CT.

### Statistics

The primary objective was to compare the rates of CNS recurrence between different treatment groups based on CNS-targeted therapy. The secondary objective was to evaluate overall survival and progression-free survival in the whole study population. Categorical variable tests were performed using two-sided Pearson chi-squared test or Fisher’s two-sided exact test, when possible. Continuous variables were analysed using the Mann-Whitney *U* test or Kruskal-Wallis test. Survival analyses with corresponding *p* values were calculated using the Kaplan-Meier method with the log-rank test. Progression-free survival was calculated from the date of the pathological diagnoses to date of disease progression, or death or the last date of follow-up. Overall survival was calculated from the date of pathological diagnosis to death or the last date of follow-up. We calculated two different CNS relapse rates. Five-year isolated CNS relapse rate included patients whose systemic disease was under control, but they experienced an isolated CNS relapse. CNS relapse rate included both isolated CNS relapses and those occurring simultaneously with a systemic relapse. CNS relapse-free survival was the time between the diagnosis date and CNS relapse. CNS survival was calculated from the CNS relapse date to death due to disease progression. Patients dying to systemic relapses were censored at the date of death. *p* values < 0.05 were considered significant. Cox regression was used to investigate the effect of several clinical factors on the risk of CNS relapse.

## Results

### Patient characteristics

The baseline demographic and clinical characteristics of all 95 patients are listed in Table 2. The male/female ratio was 1.2; overall median age was 61 ± 14 years (range 20–90). Fifty-three percent of patients had WHO ≥ 2 and the majority of patients had advanced stage disease (97%). Fifty-four percent of patients had B symptoms, 94% had IPI ≥ 3 and 48% of patients had CNS-IPI 4–6. Fifty-seven percent of patients had more than one extranodal site. Eighty-two percent of patients had an elevated LDH.

**Table 2:** Demographics and clinical characteristics at baseline

	High-risk with prophylaxis (*n* = 57, groups 1 and 2)	High-risk without prophylaxis (*n* = 38, group 3)	*p* value
Male/female ratio	1.2	1.1	0.87
Mean age (SD) Age < 60	59 (± 11) 49% (28)	70 (± 10) 21% (8)	0.006
Stage			0.034
1–2	4% (2)	3% (1)	
3–4	96% (55)	97% (37)	
WHO			0.40
0–1	51% (29)	42% (16)	
2–4	49% (28)	58% (22)	
Presence of B symptoms	46% (26)	66% (25)	0.27
IPI			
0–2	11% (6)	0% (0)	
3–5	89% (51)	100% (38)	
CNS-IPI			
0–1	5% (3)	0% (0)	
2–3	44% (25)	47% (15)	
4–6	51% (29)	53% (17)	
Extranodal sites > 1	56% (32)	58% (22)	0.87
High serum LDH level	93% (53)	66% (25)	0.001

*WHO*, World Health Organization; *IPI*, International Prognostic Index; *CNS-IPI*, Central Nervous System International Prognostic Index; *LDH*, lactate dehydrogenase; high LDH is defined as LDH over upper limit of normal

In our study population, there were four patients with testicular lymphoma. One of these patients received the treatment as per group 1 and the rest of the patients were treated as per group 2. After immunochemotherapy all these patients received contralateral scrotal irradiation.

### CNS prophylaxis

Fifty-seven patients (60%) received CNS prophylaxis. The main reasons to treat without CNS prophylaxis or to modify HD-MTX doses and frequencies were age, co-morbidities, renal insufficiency and the treating hospital. LDH level (*p* < 0.001) and age (*p* < 0.006) differed significantly between the group of patients treated without CNS-targeted treatment and the groups of patients who received CNS prophylaxis. There were no significant differences in IPI scores, CNS-IPI scores, B symptoms, WHO classification and extranodal sites between the patients receiving prophylaxis or not.

### Systemic relapse and survival

**Fig. 1:**
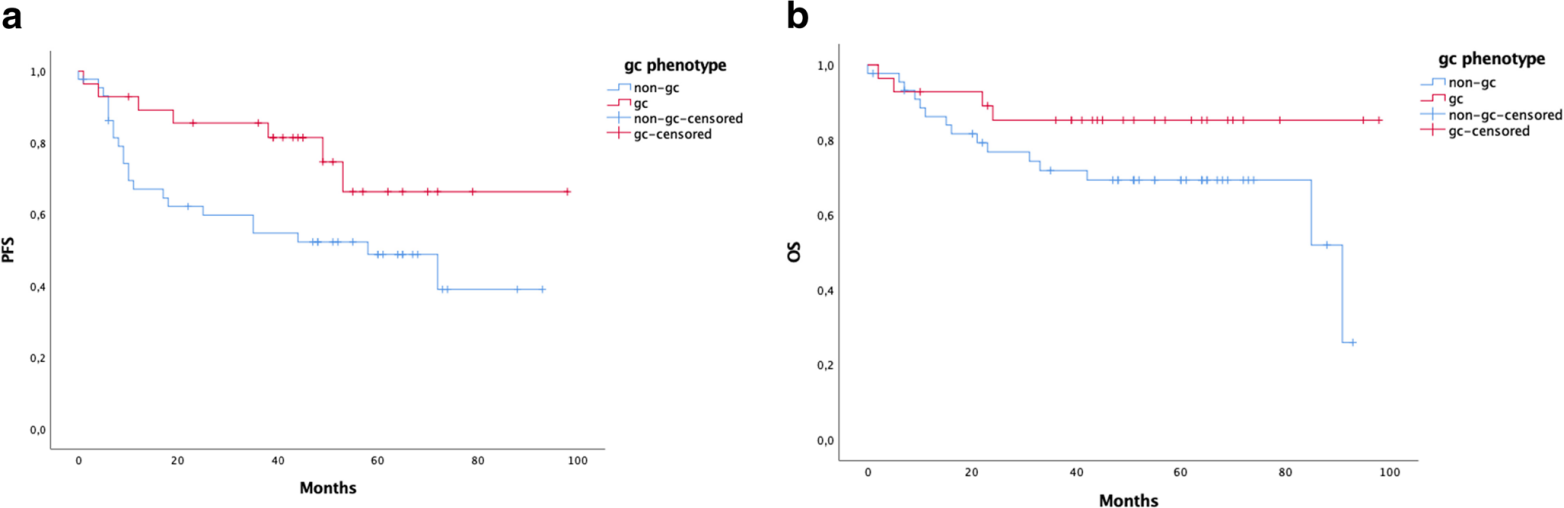
**a** Progression-free survival (PFS) (*p* = 0.044) and **b** five-year overall survival (OS) according to GC phenotypes: GCB in red and non-GC phenotype in blue

Twenty-two patients (23%) had a systemic relapse and this was diagnosed in a median time of 10 months. Median survival after the relapse was 12 months. The five-year OS in this high-risk patient population was 80% and correspondingly PFS was 63%. Five-year overall survival based on cell of origin phenotype was 85% in the germinal centre B cell type (GCB) group and 70% with activated B cell type. Corresponding rates for PFS were 66% and 49% (Fig. 1 a and b).

### CNS relapse

**Fig. 2:**
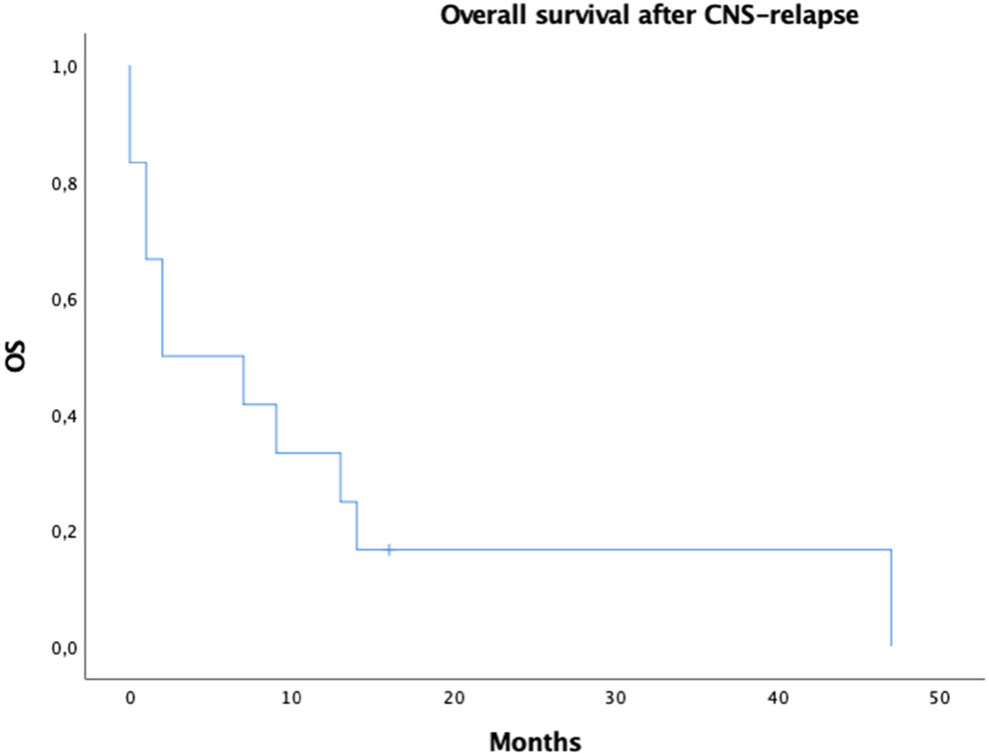
Kaplan-Meier estimate of overall survival (OS) of the patient after the detection of central nervous system (CNS) relapse

During mean follow-up time of 49 months, twelve (12.6%) CNS relapses were detected. Ten out of twelve (83%) of those were isolated in CNS system only without systemic disease. The 5-year rates of isolated CNS relapses were 5%, 10.0% and 26% (*p* = 0.034) in treatment groups 1, 2 and 3 respectively. The median time to isolated CNS relapse was 8 months (5–44 months) and the median overall survival after isolated CNS relapse was 7 months (Fig. 2).

**Fig. 3:**
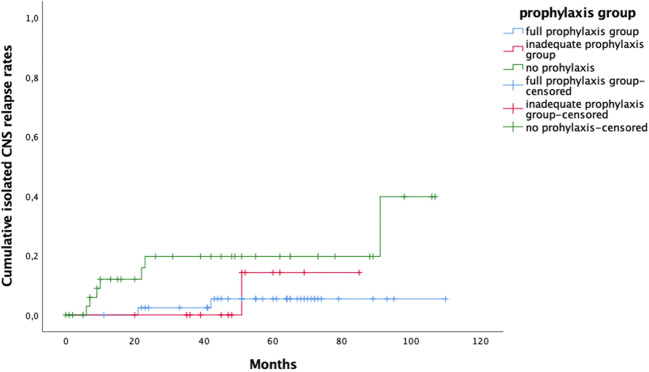
Kaplan-Meier estimate of isolated central nervous system (CNS) relapse rate based on prophylaxis group. Full prophylaxis group in blue, inadequate prophylaxis groups in red and no prophylaxis group in green

**Fig. 4:**
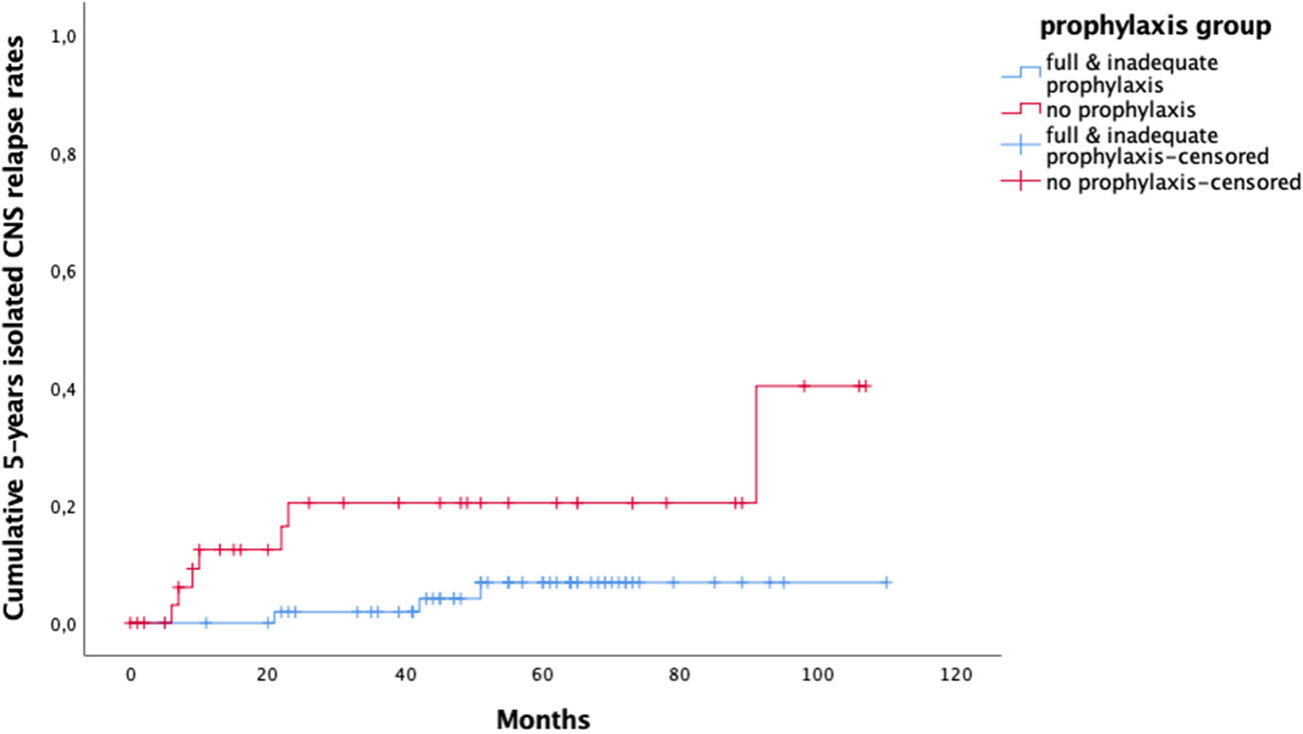
Five-year isolated CNS relapse rates based on actualized prophylaxis (*p* = 0.016). Full and inadequate prophylaxis group in blue and no prophylaxis group in red

When considering both isolated CNS relapses and those occurring simultaneously with the systemic disease, the five-year CNS relapse rates in treatment groups 1–3 were 8%, 55% and 26% respectively (Figs. 3 and 4).

### Clinicopathological factors, association with CNS relapse

Five out of twelve of the patients with CNS relapse presented with a leptomeningeal disease. Eleven out of twelve (92%) of CNS relapse patients had a non-GC phenotype. None of the patients with CNS relapse had a double hit genotype and 5/12 were presented with double expressor phenotype. None of the testicular lymphoma patients developed isolated CNS relapse, but one patient suffered a simultaneous late systemic and CNS relapse (58 months after diagnosis).

Clinical factors associated with the risk of CNS relapse were GC phenotype (*p* = 0.018) and CNS prophylaxis (*p* = 0.029). In multivariate analysis, GC phenotype and not receiving CNS prophylaxis retained their independent significance (Table 3).

**Table 3:** Clinical factors associated with the risk of CNS relapse

	*p* value	HR	95% CI Lower	Upper
Age	0.612	1.015	0.959	1.073
Prophylaxis group	0.029	4.663	1.170	18.586
GC phenotype	0.018	0.107	0.017	0.677
IPI	0.522	0.467	0.045	4.816

## Discussion

Central nervous system recurrence is a rare, but often fatal event in DLBCL. After CNS relapse, the median survival of patients is only 2–5 months. CNS-targeted therapy is an important component of treatment strategy for high-risk DLBCL patients in order to prevent this devastating complication. No consensus exists on the optimal method of CNS prophylaxis and how to define high-risk patients. In this retrospective analysis, we demonstrated that intravenous HD-MTX-containing therapy decreased the risk of CNS recurrence by 81% in a group of high-risk DLBCL patients.

The addition of rituximab to the CHOP regimen has improved treatment outcomes in DLBCL patients but had no, or only minor impact on the incidence of CNS relapse [21, 22]. The reported CNS relapse rate (5–25%) has a wide variation due to differences in clinical risk factors, study populations, accuracy of diagnostic tests and type of CNS-targeted prophylaxis [9]. The incidence of isolated CNS relapse in DLBCL without any systemic recurrence varies from 1.1 to 10.4% [23]. In this analysis, the isolated 5-year CNS relapse rate was 5% in patients with the HD-MTX-containing regimen versus 26% in patients without CNS prophylaxis. The latter number is high compared with most other published data. We find this reflects the high-risk characteristics of our lymphoma population in northern Finland, as 60% of our DLBCL patients present with high IPI characteristics (unpublished data from hospital registry).

The impact of etoposide in preventing CNS events is controversial [24–26]. In our analysis, 25/95 (26%) patients were treated with R-CHOEP and these patients were overrepresented in group 1. We cannot exclude the possibility that it might have interfered with the decrease in the CNS relapse incidence in this group.

CNS recurrence usually occurs within the first few months following diagnosis, but during the rituximab era some studies have reported that CNS relapses seem to appear later than previously [27, 28]. In line with this in the present series, median time to CNS relapse was 8 months (range 5–44 months). It also seems that, compared with pre-rituximab era, CNS relapses are more often parenchymal and isolated without systemic involvement [23, 28, 29].

Because the incidence of CNS relapse is relatively low and the toxicities of CNS prophylaxis are moderate, there is an unmet clinical need to better delineate the patients at the highest risk of CNS disease. At the moment, this selection is performed based on clinical disease presentation [30, 31] [11–13, 28, 32, 33]. IPI has remained the most commonly used tool for risk stratification. Several risk-scoring systems, which consider also some specific extra nodal involvements, have also been suggested to determine high-risk patients [11, 16, 34]. In spite of clinical presentation, lymphoma biology especially double hit genotype, non-GC phenotype or double expressor phenotype increases the risk for CNS recurrence [35–37]. In line with literature, also in the present work in multivariate analysis, non-GC phenotype and not receiving prophylaxis were independent predictors of CNS relapse.

In our study, the full CNS prophylaxis consisted of three courses of HD-MTX 3–5 g/m^2^ with or without IT MTX every three weeks on day 1 of chemoimmunotherapy cycles 1–3 or 2–4. We found a remarkable difference of the CNS relapse incidence rates between the patients treated with CNS-targeted therapy compared with patients without prophylaxis. In the full prophylaxis group, hazard ratio was 0.22. It seemed that CNS risk decreased also in the intermediate group albeit less than in the full prophylaxis group.

There is a growing evidence supporting the use of high-dose MTX as a CNS prophylaxis in DLBCL [38, 39]. Abrahamson et al. reported in a retrospective study significant reduction in the risk of CNS relapse with a median of three cycles of i.v. methotrexate 3.5 g/m^2^ administered to high-risk patients. At a median follow-up of 33 months, the incidence of CNS relapses was only 3% in the high-risk population [40]. Ferreri et al. reported a retrospective analysis of risk-tailored CNS prophylaxis in 200 DLBCL patients. One hundred seven out of two hundred patients presented with high CNS relapse risk. Thirty-three of these high-risk patients received i.v. HD-MTX prophylaxis with or without IT liposomal cytarabine and seven patients were treated only with IT. This therapy was associated with a remarkable reduction in CNS relapse rates [41].

There is also prospective data supporting this hypothesis. In a prospective Nordic phase II study (CRY-04), patients less than 65 years old with age-adjusted IPI 2 or 3 received six courses of R-CHOEP14 followed by one course of high-dose cytarabine (HD-Ara-C) and one course of HD-MTX, which resulted in a CNS relapse rate of 4.5% [25]. Most CNS relapses were detected already during chemoimmunotherapy before the planned CNS prophylaxis. For this reason in the subsequent Nordic phase II study (CHIC), systemic CNS prophylaxis with HD-MTX was given from the beginning of the therapy and CNS-targeted therapy was further intensified by intrathecally administered liposomal Ara-C. To date, interim analysis has shown 3/140 CNS relapses in a median follow-up time of 30 months (ASH abstracts 2016; dose-dense chemoimmunotherapy including early CNS prophylaxis for high-risk DLBCL. The final analysis from a Nordic phase II study (CHIC-trial) is pending). This prospective data supports the idea that delaying the CNS prophylaxis until after completing chemotherapy may result in increased CNS relapse rate [6, 42].

No consensus exists about the optimal HD-MTX dosing and frequency. But MTX at a dose of 3 g/m^2^ as 4–6 h infusion seems optimal in achieving effective concentrations and avoiding serious toxicities [43, 44]. Although some studies support some efficacy of IT chemotherapy, several others have questioned its ability to prevent CNS dissemination [45, 46].

This is a retrospective study with all the limitations and pitfalls of this setting. There were differences between patient groups in terms of age and the chemotherapy regimen selected, which may have influenced our results. However, age lost its independent prognostic value in multivariate analyses. Despite these facts, we find that our study, together with other existing data in the literature, supports the idea that high-dose methotrexate-based CNS prophylaxis is associated with a significant reduction in CNS relapse rates. We recommend that it should be considered in DLBCL patients at high risk for CNS recurrence. At the moment, we cannot draw definitive conclusions about the optimal number of MTX courses. Further investigations are necessary to better understand the optimal dose, quantity and timing of high-dose systemic methotrexate required to achieve optimal prophylaxis and the possible additional effect of IT MTX. Randomized clinical trials and prospective studies are required to confirm these findings. Also there is a need for finding better prognostic models, biological factors or imaging techniques to define the real high-risk patient population.

## References

[1] Campo E, Swerdlow SH, Harris NL, Pileri S, Stein H, Jaffe ES (2011). The 2008 WHO classification of lymphoid neoplasms and beyond: evolving concepts and practical applications. Blood.

[2] Martelli M, Ferreri AJ, Agostinelli C, Di Rocco A, Pfreundschuh M, Pileri SA (2013). Diffuse large B-cell lymphoma. Crit Rev Oncol Hematol.

[3] Feugier P, Van Hoof A, Sebban C, Solal-Celigny P, Bouabdallah R, Ferme C (2005). Longterm results of the R-CHOP study in the treatment of elderly patients with diffuse large B-cell lymphoma: a study by the Groupe d'Etude des Lymphomes de l'Adulte. J Clin Oncol.

[4] Pfreundschuh M, Trumper L, Osterborg A, Pettengell R, Trneny M, Imrie K (2006). CHOPlike chemotherapy plus rituximab versus CHOP-like chemotherapy alone in young patients with good-prognosis diffuse large-B-cell lymphoma: a randomised controlled trial by the MabThera International Trial (MInT) Group. Lancet Oncol.

[5] Coiffier B, Lepage E, Briere J, Herbrecht R, Tilly H, Bouabdallah R (2002). CHOP chemotherapy plus rituximab compared with CHOP alone in elderly patients with diffuse large-B-cell lymphoma. N Engl J Med.

[6] Bernstein SH, Unger JM, Leblanc M, Friedberg J, Miller TP, Fisher RI (2009). Natural history of CNS relapse in patients with aggressive non-Hodgkin’s lymphoma: a 20-year follow-up analysis of SWOG 8516 -- the Southwest Oncology Group. J Clin Oncol.

[7] Boehme V, Schmitz N, Zeynalova S, Loeffler M, Pfreundschuh M (2009). CNS events in elderly patients with aggressive lymphoma treated with modern chemotherapy (CHOP-14) with or without rituximab: an analysis of patients treated in the RICOVER-60 trial of the German High-Grade Non-Hodgkin Lymphoma Study Group (DSHNHL). Blood.

[8] Feugier P, Virion JM, Tilly H, Haioun C, Marit G, Macro M, Bordessoule D, Recher C, Blanc M, Molina T, Lederlin P, Coiffier B (2004). Incidence and risk factors for central nervous system occurrence in elderly patients with diffuse large-B-cell lymphoma: influence of rituximab. Ann Oncol.

[9] Bierman P, Giglio P. Diagnosis and treatment of central nervous system involvement in non-Hodgkin’s lymphoma. Hematol Oncol Clin North Am 2005 August 01;19(4):609, v 10.1016/j.hoc.2005.05.003 16083825

[10] Hegde U, Filie A, Little RF, Janik JE, Grant N, Steinberg SM (2005). High incidence of occult leptomeningeal disease detected by flow cytometry in newly diagnosed aggressive B cell lymphomas at risk for central nervous system involvement: the role of flow cytometry versus cytology. Blood.

[11] Hollender A, Kvaloy S, Nome O, Skovlund E, Lote K, Holte H (2002). Central nervous system involvement following diagnosis of non-Hodgkin’s lymphoma: a risk model. Ann Oncol.

[12] van Besien K, Ha CS, Murphy S, McLaughlin P, Rodriguez A, Amin K, Forman A, Romaguera J, Hagemeister F, Younes A, Bachier C, Sarris A, Sobocinski KS, Cox JD, Cabanillas F (1998). Risk factors, treatment, and outcome of central nervous system recurrence in adults with intermediate-grade and immunoblastic lymphoma. Blood.

[13] Zinzani PL, Magagnoli M, Frezza G, Prologo G, Gherlinzoni F, Bendandi M, Albertini P, Babini L, D’alessandro R, Tura S (1999). Isolated central nervous system relapse in aggressive non-Hodgkin’s lymphoma: the Bologna experience. Leuk Lymphoma.

[14] Herrlinger U, Glantz M, Schlegel U, Gisselbrecht C, Cavalli F (2009). Should intracerebrospinal fluid prophylaxis be part of initial therapy for patients with non-Hodgkin lymphoma: what we know, and how we can find out more. Semin Oncol.

[15] Shipp M, Harrington D, Anderson J, Armitage J, Bonadonna G, Brittinger G (1993). A predictive model for aggressive non-Hodgkin’s-lymphoma. N Engl J Med.

[16] Schmitz N, Zeynalova S, Nickelsen M, Kansara R, Villa D, Sehn LH, Glass B, Scott DW, Gascoyne RD, Connors JM, Ziepert M, Pfreundschuh M, Loeffler M, Savage KJ (2016). CNS International Prognostic Index: a risk model for CNS relapse in patients with diffuse large B-cell lymphoma treated with R-CHOP. J Clin Oncol.

[17] Carbone PP, Kaplan HS, Musshoff K, Smithers DW, Tubiana M (1971). Report of the committee on Hodgkin’s disease staging classification. Cancer Res.

[18] Hans CP, Weisenburger DD, Greiner TC, Gascoyne RD, Delabie J, Ott G, Müller-Hermelink HK, Campo E, Braziel RM, Jaffe ES, Pan Z, Farinha P, Smith LM, Falini B, Banham AH, Rosenwald A, Staudt LM, Connors JM, Armitage JO, Chan WC (2004). Confirmation of the molecular classification of diffuse large B-cell lymphoma by immunohistochemistry using a tissue microarray. Blood.

[19] Cheson BD, Horning SJ, Coiffier B, Shipp MA, Fisher RI, Connors JM (1999). Report of an international workshop to standardize response criteria for non-Hodgkin’s lymphomas. NCI Sponsored International Working Group. J Clin Oncol.

[20] Cheson BD, Pfistner B, Juweid ME, Gascoyne RD, Specht L, Horning SJ, Coiffier B, Fisher RI, Hagenbeek A, Zucca E, Rosen ST, Stroobants S, Lister TA, Hoppe RT, Dreyling M, Tobinai K, Vose JM, Connors JM, Federico M, Diehl V, International Harmonization Project on Lymphoma (2007). Revised response criteria for malignant lymphoma. J Clin Oncol.

[21] Tomita N, Yokoyama M, Yamamoto W, Watanabe R, Shimazu Y, Masaki Y, Tsunoda S, Hashimoto C, Murayama K, Yano T, Okamoto R, Kikuchi A, Tamura K, Sato K, Sunami K, Shibayama H, Takimoto R, Ohshima R, Hatta Y, Moriuchi Y, Kinoshita T, Yamamoto M, Numata A, Ishigatsubo Y, Takeuchi K (2012). Central nervous system event in patients with diffuse large B-cell lymphoma in the rituximab era. Cancer Sci.

[22] Ghose A, Elias HK, Guha G, Yellu M, Kundu R, Latif T (2015). Influence of rituximab on central nervous system relapse in diffuse large B-cell lymphoma and role of prophylaxis--a systematic review of prospective studies. Clin Lymphoma Myeloma Leuk.

[23] Villa D, Connors JM, Shenkier TN, Gascoyne RD, Sehn LH, Savage KJ (2010). Incidence and risk factors for central nervous system relapse in patients with diffuse large B-cell lymphoma: the impact of the addition of rituximab to CHOP chemotherapy. Ann Oncol.

[24] Boehme V, Zeynalova S, Kloess M, Loeffler M, Kaiser U, Pfreundschuh M, Schmitz N, German High-Grade Non-Hodgkin’s Lymphoma Study Group (DSHNHL) (2007). Incidence and risk factors of central nervous system recurrence in aggressive lymphoma--a survey of 1693 patients treated in protocols of the German High-Grade Non-Hodgkin’s Lymphoma Study Group (DSHNHL). Ann Oncol.

[25] Holte H, Leppa S, Bjorkholm M, Fluge O, Jyrkkio S, Delabie J (2013). Dose-densified chemoimmunotherapy followed by systemic central nervous system prophylaxis for younger high-risk diffuse large B-cell/follicular grade 3 lymphoma patients: results of a phase II Nordic Lymphoma Group study. Ann Oncol.

[26] Schmitz N, Zeynalova S, Glass B, Kaiser U, Cavallin-Stahl E, Wolf M, Haenel M, Loeffler M, Truemper L, Pfreundschuh M (2012). CNS disease in younger patients with aggressive B-cell lymphoma: an analysis of patients treated on the Mabthera International Trial and trials of the German High-Grade Non-Hodgkin Lymphoma Study Group. Ann Oncol.

[27] Guirguis HR, Cheung MC, Mahrous M, Piliotis E, Berinstein N, Imrie KR, Zhang L, Buckstein R (2012). Impact of central nervous system (CNS) prophylaxis on the incidence and risk factors for CNS relapse in patients with diffuse large B-cell lymphoma treated in the rituximab era: a single centre experience and review of the literature. Br J Haematol.

[28] Shimazu Y, Notohara K, Ueda Y (2009). Diffuse large B-cell lymphoma with central nervous system relapse: prognosis and risk factors according to retrospective analysis from a single-center experience. Int J Hematol.

[29] Kumar A, Vanderplas A, LaCasce AS, Rodriguez MA, Crosby AL, Lepisto E (2012). Lack of benefit of central nervous system prophylaxis for diffuse large B-cell lymphoma in the rituximab era: findings from a large national database. Cancer.

[30] Zucca E, Conconi A, Mughal TI, Sarris AH, Seymour JF, Vitolo U, Klasa R, Ozsahin M, Mead GM, Gianni MA, Cortelazzo S, Ferreri AJ, Ambrosetti A, Martelli M, Thiéblemont C, Moreno HG, Pinotti G, Martinelli G, Mozzana R, Grisanti S, Provencio M, Balzarotti M, Laveder F, Oltean G, Callea V, Roy P, Cavalli F, Gospodarowicz MK, International Extranodal Lymphoma Study Group (2003). Patterns of outcome and prognostic factors in primary large-cell lymphoma of the testis in a survey by the International Extranodal Lymphoma Study Group. J Clin Oncol.

[31] Kridel R, Telio D, Villa D, Sehn LH, Gerrie AS, Shenkier T, Klasa R, Slack GW, Tan K, Gascoyne RD, Connors JM, Savage KJ (2017). Diffuse large B-cell lymphoma with testicular involvement: outcome and risk of CNS relapse in the rituximab era. Br J Haematol.

[32] Keldsen N, Michalski W, Bentzen SM, Hansen KB, Thorling K (1996). Risk factors for central nervous system involvement in non-Hodgkins-lymphoma--a multivariate analysis. Acta Oncol.

[33] Sehn LH, Scott DW, Chhanabhai M, Berry B, Ruskova A, Berkahn L, Connors JM, Gascoyne RD (2011). Impact of concordant and discordant bone marrow involvement on outcome in diffuse large B-cell lymphoma treated with R-CHOP. J Clin Oncol.

[34] Kanemasa Y, Shimoyama T, Sasaki Y, Tamura M, Sawada T, Omuro Y (2016). Central nervous system relapse in patients with diffuse large B cell lymphoma: analysis of the risk factors and proposal of a new prognostic model. Ann Hematol.

[35] Aukema SM, Siebert R, Schuuring E, van Imhoff GW, Kluin-Nelemans HC, Boerma EJ, Kluin PM (2011). Double-hit B-cell lymphomas. Blood.

[36] Savage KJ, Slack GW, Mottok A, Sehn LH, Villa D, Kansara R, Kridel R, Steidl C, Ennishi D, Tan KL, Ben-Neriah S, Johnson NA, Connors JM, Farinha P, Scott DW, Gascoyne RD (2016). Impact of dual expression of MYC and BCL2 by immunohistochemistry on the risk of CNS relapse in DLBCL. Blood.

[37] Li S, Lin P, Fayad LE, Lennon PA, Miranda RN, Yin CC, Lin E, Medeiros LJ (2012). B-cell lymphomas with MYC/8q24 rearrangements and IGH@BCL2/t(14;18)(q32;q21): an aggressive disease with heterogeneous histology, germinal center B-cell immunophenotype and poor outcome. Mod Pathol.

[38] Bokstein F, Lossos A, Lossos IS, Siegal T (2002). Central nervous system relapse of systemic non-Hodgkin’s lymphoma: results of treatment based on high-dose methotrexate combination chemotherapy. Leuk Lymphoma.

[39] Cheah CY, Herbert KE, O’Rourke K, Kennedy GA, George A, Fedele PL, Gilbertson M, Tan SY, Ritchie DS, Opat SS, Prince HM, Dickinson M, Burbury K, Wolf M, Januszewicz EH, Tam CS, Westerman DA, Carney DA, Harrison SJ, Seymour JF (2014). A multicentre retrospective comparison of central nervous system prophylaxis strategies among patients with high-risk diffuse large B-cell lymphoma. Br J Cancer.

[40] Abramson JS, Hellmann M, Barnes JA, Hammerman P, Toomey C, Takvorian T, Muzikansky A, Hochberg EP (2010). Intravenous methotrexate as central nervous system (CNS) prophylaxis is associated with a low risk of CNS recurrence in high-risk patients with diffuse large B-cell lymphoma. Cancer.

[41] Ferreri AJ, Bruno-Ventre M, Donadoni G, Ponzoni M, Citterio G, Foppoli M (2015). Risk-tailored CNS prophylaxis in a mono-institutional series of 200 patients with diffuse large B-cell lymphoma treated in the rituximab era. Br J Haematol.

[42] Villa D, Connors JM, Sehn LH, Gascoyne RD, Savage KJ (2011). Diffuse large B-cell lymphoma with involvement of the kidney: outcome and risk of central nervous system relapse. Haematologica.

[43] Ferreri AJ, Guerra E, Regazzi M, Pasini F, Ambrosetti A, Pivnik A (2004). Area under the curve of methotrexate and creatinine clearance are outcome-determining factors in primary CNS lymphomas. Br J Cancer.

[44] Brugieres L, Le Deley MC, Rosolen A, Williams D, Horibe K, Wrobel G (2009). Impact of the methotrexate administration dose on the need for intrathecal treatment in children and adolescents with anaplastic large-cell lymphoma: results of a randomized trial of the EICNHL Group. J Clin Oncol.

[45] Arkenau HT, Chong G, Cunningham D, Watkins D, Agarwal R, Sirohi B, Trumper M, Norman A, Wotherspoon A, Horwich A (2007). The role of intrathecal chemotherapy prophylaxis in patients with diffuse large B-cell lymphoma. Ann Oncol.

[46] Chua SL, Seymour JF, Streater J, Wolf MM, Januszewicz EH, Prince HM (2002). Intrathecal chemotherapy alone is inadequate central nervous system prophylaxis in patients with intermediate-grade non-Hodgkin’s lymphoma. Leuk Lymphoma.

